# Partial intraoperative signal recovery is associated with normal postoperative vocal cord motility in patients with intraoperative loss of signal

**DOI:** 10.1007/s13304-025-02373-0

**Published:** 2025-08-20

**Authors:** Pierpaolo Gallucci, Priscilla Francesca Procopio, Francesco Pennestrì, Giuseppe Marincola, Lucia D’Alatri, Annamaria Martullo, Carmela De Crea, Marco Raffaelli

**Affiliations:** 1 UOC Chirurgia Endocrina E Metabolica, Fondazione Policlinico Universitario Agostino Gemelli IRCCS, L.Go Agostino Gemelli 8, 00168 Rome, Italy; 2 Centro Di Ricerca in Chirurgia Delle Ghiandole Endocrine E Dell’Obesità, Università Cattolica del Sacro Cuore, Rome, Italy; 3 UOC Otorinolaringoiatria, Fondazione Policlinico Universitario Agostino Gemelli IRCCS, Rome, Italy

**Keywords:** Thyroidectomy, Loss of signal, Intraoperative nerve neuromonitoring (IOMN), Intraoperative signal recovery, Vocal fold palsy

## Abstract

Loss of signal (LOS) at intraoperative nerve monitoring (IONM) is defined as an >100 mV amplitude decrease  and a >10% latency reduction and represents a predictor of postoperative impaired vocal cord motility (VCM). We aimed to evaluate if an intraoperative signal recovery (ISR) after LOS may predict a positive outcome of VCM. Among 5884 consecutive intermittent IONM-guided thyroidectomies (April 2021- March 2025) all the patients in whom a LOS was observed were evaluated. Topic and intravenous corticosteroids were administered to all of them. Eventual recovery was evaluated after 20 minutes. Patients with  an ISR less than  50% compared to the baseline were included. The rate of vagal signal (VS) ISR was defined as a percent from the minimum value: VS-recovery–VS-minimal/VS-predissection–VS-minumum. ISR was correlated to VCM (ROC curve analysis). Among 169 patients with LOS, 65 (38.5%) showed ISR, with 48 (73.8%) of them exhibiting normal VCM on postoperative day 1 (POD-1). The remaining 17 patients with impaired VCM on POD-1 recovered normal VCM on POD-15 (7–10.8%) or POD-30 (10–15.4%). The AUC for impaired VCM at POD-1 was 0.938 (95% CI: 0.849–0.983, p <0.0001) and the ISR cut-off was 13%, with a 94.1% sensitivity and a 89.6% specificity. All patients with ISR >31% showed normal VCM. All patients with ISR <13% exhibited impaired motility at POD-15 but recovered at POD-30. ISR can predict full recovery of VCM. ISR >31% is associated with normal postoperative VCM and staged thyroidectomy could be avoided in this subgroup of patients with LOS.

## Introduction

Although vocal cord paralysis (VCP) is not the most frequent complication following thyroid surgery, it still represents perhaps the most feared outcome, serving as a primary cause of medico-legal issues[1–3]. Specifically, inferior laryngeal nerve (ILN) palsy can severely impact on patients’ quality of life, leading to an increased risk of postoperative dysphagia, dysphonia, and less frequently even aspiration pneumonia[4]. In more severe cases, bilateral damage to the nerve may lead to life-threatening complications, necessitating tracheostomy in approximately one-third of affected patients[5].

In this view, in recent decades, intraoperative neuromonitoring (IONM) has been introduced with the aim of reducing the incidence of these complications and facilitating their early identification[6, 7]. Thereafter, IONM-assisted thyroidectomy has progressively become of routinary use during endocrine surgeries.

The prompt identification of loss of signal (LOS) of ILN during surgery by means of IONM provides the opportunity for early prediction of alterations in vocal cord motility (VCM) in the postoperative setting[8]. Moreover, IONM is an essential tool for the reduction of the risk of bilateral VCP, enabling safe staged surgeries in cases of planned total thyroidectomy (TT) when LOS occurs on the first side of surgical dissection[9]. In this regard, the clinical relevance of staged thyroidectomy (ST) gained increasing prominence in recent years[10–12], with this surgical approach being employed even in cases of malignant and advanced diseases, as well as in conditions related to hyperfunction, all with the explicit aim of avoiding the potential life-threating consequences of bilateral ILN paralysis[13].

Various authors concerted their efforts to identify potential risk factors for LOS[14, 15] and to evaluate the impact of the type of LOS on the occurrence of postoperative VCP[16]. In this context, the accurate detection of VCM-related pre- and intraoperative issues can significantly decrease the risk of VCP and facilitate timely treatment for any functional impairments, thereby promoting recovery.

Noteworthy, not all the intraoperative adverse events affecting the ILN fall under the category of complete LOS. If LOS may remain complete after surgery, in some cases a phenomenon referred to as "intraoperative recovery after LOS" may also be observed[17]. In these circumstances, LOS is followed by the registration of partial intraoperative signal recovery (ISR) and functional healing primarily depends on the degree of amplitude recovery[16]. Delving deeper, favourable prognostic factors for functional healing are represented by the registration, after LOS, of signal recovery with a >50% amplitude curve or with <10% latency, compared to the baseline values. Conversely, inferior clinical outcomes are observed in those cases whether signal recovery amplitude is less than 50% and latency greater than 10%, although precise cut-off of ISR, which may serve as a predictive factor for postoperative laryngeal impairment, has not been assessed yet[16].

The present study aims to evaluate the optimal cut-off for ISR to predict normal vocal fold motility, evaluated with postoperative flexible fiberoptic laryngoscopy, in patients who experienced LOS and less than 50% ISR. These results may lead to the correct identification of those patients which may avoid ST, enabling more tailored and effective surgical strategies.

## Methods

This retrospective analysis was conducted on patients who underwent thyroidectomy with intermittent IONM guidance. A total of 5884 thyroid procedures, comprising 7538 nerves at risk (NAR), were performed between April 2021 and March 2025 at our referral center of endocrine surgery. Patients' demographics and clinical characteristics are summarized in Table 1.

**Table 1:** Population’s characteristics

Number of patients	5884
Age, years (mean ± SD)	50.3 ± 16.1
BMI, kg/m^2^ (mean ± SD)	24.9 ± 3.8
Sex (M/F)	1824 (31%)/4060 (69%)
Malignant/Benign	3324 (56.5%)/2560 (43.5%)
*Surgical procedure*	
TL	588 (10%)
TT	3295 (56%)
TT + ND	1883 (32%)
Reoperation	118 (2%)
Nerves at risk	7538
*Hypoparathyroidism*	
Transient	1377 (26%)
Permanent	79 (1.5%)

*TT* Total thyroidectomy, *TL* Lobectomy, *CND* Central neck dissection (unilateral or bilateral), *ND* neck dissection (central and/or lateral)

### Inclusion and exclusion criteria

**Fig. 1:**
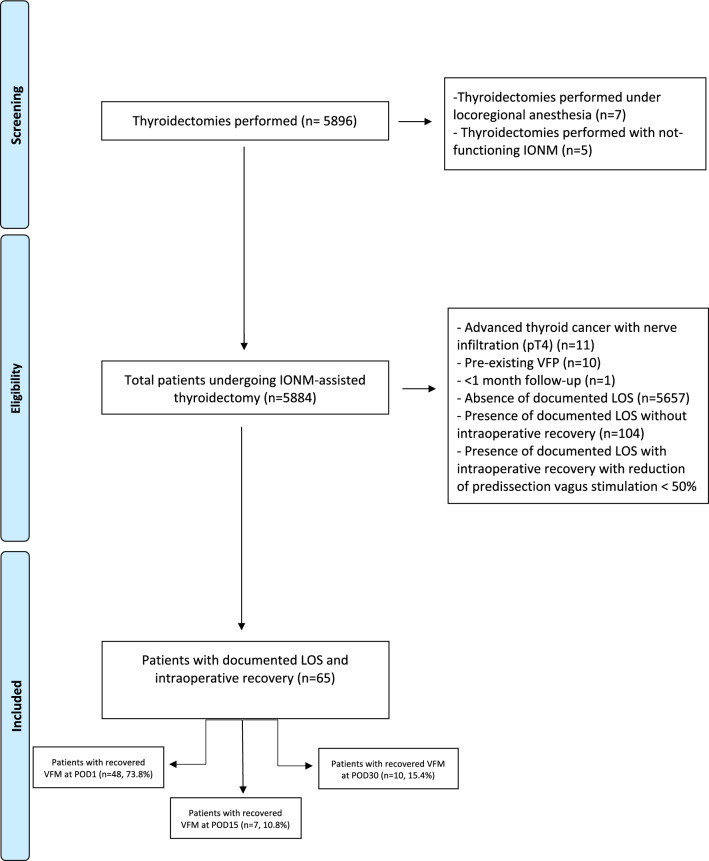
Patient selection process: Flow-chart diagram

The inclusion criteria encompassed all patients who, following a LOS, exhibited an ISR that was not significant, remaining below 50% of the predissection vagal stimulation, after intraoperative administration of topic and intravenous corticosteroids. To be included, patients required complete IONM data, as well as documented pre- and postoperative vocal cord evaluation, and adherence to follow-up schedules. This ensured a comprehensive analysis of outcomes related to VCM. Patients were excluded from the study whether they experienced complete LOS with intraoperative LOS persistence, complete signal recovery, or final ISR>50%. Additionally, those patients with incomplete IONM or follow-up data, pre-existing  VCP, or advanced thyroid malignancies withILN infiltration were also excluded (Fig. 1). This stringent selection process aimed to create a homogeneous patients' population for the analysis.

### Patient demographics and surgical details

Patients' demographic data included age, sex as assigned at birth, and body mass index (BMI). The recorded surgical details encompassed the type of procedure, which included total thyroidectomy (TT), lobectomy (TL), total thyroidectomy with neck dissection (TT + ND), and reoperations. Additionally, the number of NARs per patient was documented to analyze the extent of nerve monitoring performed. Clinical characteristics, such as whether the underlying condition was benign or malignant, were documented, along with preoperative diagnoses, which included benign goiters, toxic goiters, and suspicious nodules. This information provided insight into the nature of the conditions being treated and how they might relate to surgical outcomes. Postoperative complications were noted, particularly focusing on the incidence of transient and permanent hypoparathyroidism, which are significant concerns following thyroid surgery.

### Intraoperative procedures and monitoring

In our study, all the guidelines from the International Neural Monitoring Study Group (INMSG) were adopted and followed [18]; however, the stimulation level was set at 0.5 mA as a precautionary measure.

In our clinical practice, a non-invasive neuromonitoring was used, which implies the positioning of an endotracheal tube designed to convert vocal cord movements into electrical impulses. Moreover, our experience was limited to the use of intermittent IONM (I-IONM), due to the unavailability of continuous IONM (C-IONM) in our Institution during the study period, with the characteristics and advantages of each one being well-documented in literature[19]. 

The anaesthesiologic protocol implies the administration, during the induction part of anesthesia, of 1.5- 2 mg/Kg Propofol, 3–5 mcg/Kg Fentanyl and 10–20 mg of Rocuronium bromide, in order to permit the correct IONM. During the surgical procedure, 0.05–0.15 mcg/Kg/min Remifentanil is administered, as well as Sevoflurane (until bispectral index-BIS values of 40–60).

All surgeries employed standardized IONM protocols to monitor vagal nerve amplitude and latency changes before and after thyroid dissection. IONM was performed using the C2 Xplore system (Inomed Medizintechnik GmbH, Emmendingen, Germany). A reduction in amplitude greater than 50% after LOS was considered significant, indicating a potential compromise in nerve function. After LOS, the protocol necessitated the administration of topic and intravenous corticosteroids to mitigate inflammation and promote nerve recovery. A waiting period of 20 minutes was observed, during which if an initial recovery of the action potential was recorded, corticosteroids were again administered topically. Following an additional 20 minutes, new stimulation was conducted to assess recovery progress. ISR was defined as a measurable improvement in vagal signal amplitude that remained below 50% of the predissection baseline values. This definition underscores the importance of monitoring not just recovery but also the degree of recovery in relation to baseline function. The ISR rate was calculated using the formula:

$$ISR \, rate = \frac{{VS \, {amplitude} \, {after} \, {recovery} - {VS} \, {amplitude} \,  {minimum}}}{VS \, {amplitude} \, {pre} - {dissection} \, - \, {VS} \, {amplitude} \, {minimum}}$$

This formula allows for a quantifiable measure of signal recovery that can be correlated with the postoperative outcomes.

Corticosteroid treatment with methylprednisolone 16 mg was performed in those patients who did not show VCM recovery in POD-1, according to the following protocol: one administration per day for 4 days, half administration per day for the following 4 days, and half administration for additional 3 doses.

### Postoperative evaluation

Postoperative VCM was systematically evaluated at multiple time points, starting on POD-1. Patients who exhibited impaired VCM on POD-1 underwent further assessments on POD-15 and POD-30 to monitor recovery trends over time. VCM recovery was defined as the return to normal vocal fold motion at any of these postoperative intervals, reflecting the effectiveness of intraoperative monitoring and postoperative care. Postoperative VCM assessment was performed using flexible fiberoptic laryngoscopy. A 3.4-mm fiberoptic rhinolaryngoscope connected to a video processor (XION GmbH, Germany) with dedicated processing software was utilized for these evaluations. The assessment focused on the mobility and positioning of the vocal folds, as well as arytenoid movement during phonation and respiration. All laryngoscopy procedures were conducted by the same experienced otolaryngologist to ensure consistency and reliability of the findings. This uniformity in evaluation is crucial for minimizing variability in results. Definition of transient/permanent nerve palsy[20] and transient[21]/persistent[22] hypoparathyroidism has been previously reported.

### Endpoints

The primary endpoint of this study consisted in the correlation between ISR rates and VCM recovery, with a specific focus on identifying a predictive ISR threshold for recovery on POD-1. The secondary endpoints included an analysis of the sensitivity, specificity, and predictive values associated with varying ISR rates. Comparisons were made between different patients' groups stratified by ISR thresholds, and recovery proportions were calculated at each follow-up interval, adding depth to the analysis of recovery patterns.

### Ethical considerations

This study adhered to the Declaration of Helsinki and received approval from the institutional review board. Written informed consent was obtained from all patients included in the study, ensuring ethical compliance and respect for patients' autonomy.

### Statistical analysis

Categorical variables were summarized as absolute numbers (n) and percentages (%), providing a clear overview of the patient population. Continuous variables were presented as means (m) and standard deviations (SD) to facilitate comparisons. The Shapiro–Wilk test was employed to assess the normality of the data. Parametric tests were applied to variables with a normal distribution, while non-parametric tests were utilized for those without. A receiver operating characteristic (ROC) curve analysis was conducted to evaluate the predictive accuracy of ISR thresholds. The area under the curve (AUC) was calculated to assess diagnostic performance, and the optimal ISR cutoff for predicting recovery on POD-1 and POD-15 was identified. Sensitivity and specificity were reported for the selected threshold to provide a comprehensive understanding of the predictive power of ISR. A P-value of less than 0.05 was considered statistically significant, indicating meaningful results that could impact clinical practice. Statistical analysis was performed using IBM SPSS Statistics for Windows, Version 25.0 (Armonk, NY: IBM Corp).

## Results

This retrospective analysis included patients who underwent thyroidectomy between April 2021 and March 2025. During this period, a total of 5896 thyroid procedures (7538 NAR) were performed; of these, 5884 procedures, corresponding to the study population, were conducted under I-IONM.

Table 1 summarizes the baseline characteristics of these 5884 monitored patients. The mean age of patients was 50.3 ± 16.1 years, with a mean BMI of 24.9 ± 3.8 kg/m^2^. Females accounted for the majority (4060 vs. 1824 males), highlighting a gender disparity in thyroid surgery patients. Diagnoses were malignant in 3324 (56.5%) cases and benign in 2560 (43.5%) cases, indicating a significant prevalence of malignancy in this cohort. Surgical interventions included TT in 3295 (56%) cases , TL in 588 (10%) cases, TT + ND in 1883 (32%) cases, and reoperations in 118 (2%) cases. The total number of NARs was 7538, reflecting the comprehensive nerve monitoring conducted throughout the surgical procedures.

**Fig. 2:**
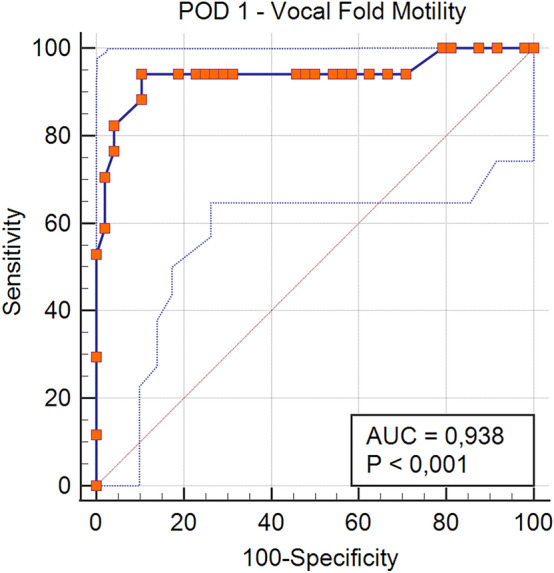
ROC Curve of ISR values to predict VFM on POD-1

**Fig. 3:**
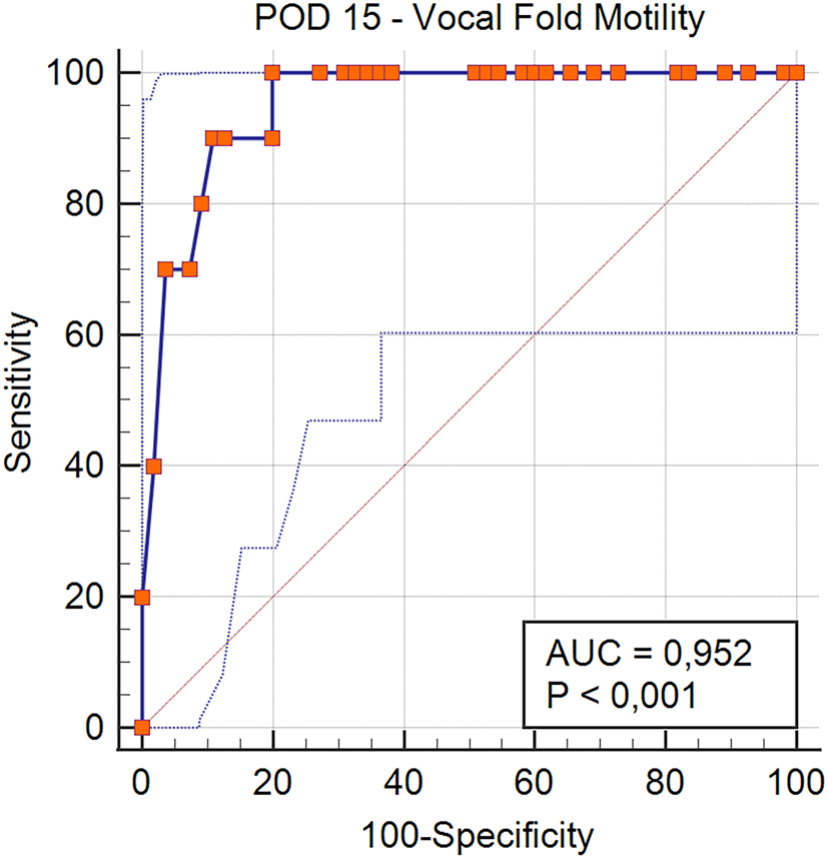
ROC Curve of ISR values to predict VFM on POD-15

Table 2 presents characteristics of a subgroup of 65 patients who demonstrated partial ISR. These patients had a mean age of 54.9 ± 12.9 years and a mean BMI of 26 ± 5.1 kg/m^2^. Females represented the majority (45 vs. 20 males), which is consistent with the broader trends observed in thyroid surgery demographics. Preoperative diagnoses included benign conditions in 25 patients, toxic goiter in 4 patients, and suspicious nodules in 36 patients. Surgical procedures consisted of TL in 13 (20%) cases, TT in 27 (41.5%) cases, TT + ND in 21 (32.3%) cases, and reoperation in 4 (6.2%) cases. The total number of NARs in this subgroup was 113, which underscores the complexity of the cases being managed. Among the 65 patients (113 NARs) included in the study that showed ISR, 48 (73.8%) patients showed normal VCM on POD-1. The remaining 17 patients with impaired POD-1 VCM recovered normal VCM on POD-15 (7–10.8%) or POD-30 (10–15.4%), indicating a positive trend in recovery over time. In only one case, the ISR following LOS occurred on the first side of surgical dissection, prompting a modification in the therapeutic strategy (two-stage thyroidectomy). Completion thyroidectomy was performed 3 months after the index procedure, following documented recovery of VCM at POD-30, due to pathological detection of thyroid cancer with intermediate ATA risk features [23] and basing on the patient’s preference. This case exemplifies the importance of individualized treatment plans in managing complex thyroid conditions. Transient and definitive hypoparathyroidism were detected in 33.8% and 1.5% of patients, respectively, highlighting the importance of monitoring these complications in the postoperative period. The mean percentage of maximum signal reduction between the predissection vagal nerve value (V1) and the first stimulated post-dissection vagal nerve value (V2min) was 82.2%, with a minimum value of 52.4% and a maximum value of 99.4%. This significant reduction underscores the challenges faced during thyroid surgery and the importance of effective monitoring techniques. The mean percentage of maximum signal reduction between V1 and the last stimulated post-dissection vagal nerve value (V2max) was 69.6%, with a minimum value of 51.7% and a maximum value of 94.2%. ROC curve analysis at POD-1 showed an AUC of 0.938 (95% CI: 0.849–0.983, p < 0.0001), indicating excellent predictive accuracy (Fig. 2). The optimal Youden index was 0.837, corresponding to an ISR cut-off value of ≤ 0.13, yielding a sensitivity of 94.1% and specificity of 89.6%. The lowest ISR value at POD-1 ensuring subsequent recovery of VCM was 0.31, associated with a sensitivity of 100%, but with relatively low specificity (20.8%), indicating a considerable rate of false positives at this threshold. At POD-15, ROC analysis showed an AUC of 0.952 (95% CI: 0.868–0.989, p < 0.0001), further confirming the robustness of the ISR thresholds (Fig. 3). The Youden index reached a maximum of 0.800, also at an ISR cut-off value of ≤ 0.13, with a sensitivity of 100% and specificity of 80%. At the ISR value of 0.31, sensitivity remained 100%, but specificity slightly decreased to 18.2%, underscoring the conservative nature of this threshold for predicting normal postoperative VCM. All the patients who did not achieve adequate recovery exhibited vocal cord hypomotility on POD-1; however, complete paralysis on POD-1 was never observed, suggesting that while patients may experience varying degrees of impairment, full paralysis was uncommon in this cohort. ISR ≤ 31% was related with a 100% specificity for VCP in POD-1 (50% sensitivity).

**Table 2:** Characteristics of the included patients with intraoperative signal recovery

Number of patients	65
Age, years (mean ± SD)	54.9 ± 12.9
BMI, kg/m^2^ (mean ± SD)	26 ± 5.1
Sex (M/F)	20/45
*Preoperative diagnosis*	
Benign	25 (38.5%)
Toxic goiter	4 (6.1%)
Suspicious nodule	36 (55.4%)
*Surgical procedure*	
TL	13 (20%)
TT	27 (41.5%)
TT + ND	21 (32.3%)
Reoperation	4 (6.2%)
Nerves at risk	113
*Hypoparathyroidism*	
Transient	22 (33.8%)
Permanent	1 (1.5%)

*TT* Total thyroidectomy, *TL* Lobectomy, *CND* Central neck dissection (unilateral or bilateral), *ND* neck dissection (central and/or lateral), *POD* Postoperative Day

## Discussion

This study supports the prognostic value of the impact of ISR on postoperative functional recovery even in cases of signal reduction exceeding 50% compared to predissection values.

Indeed, according to our results, the detection of ISR ≥ 31% between the minimal and the maximal values of signal recovery following LOS has been related to normal VCM in POD-1. Moreover, all patients who experienced intraoperative recovery, regardless from the values, present normal VCM within 30 days from the surgical procedure.

The relevance of these findings relies on the potential for appropriate modulation of surgical strategy, which may help to avoid ST when specific intraoperative recovery parameters are documented.

If the type of LOS is known to be predictive of postoperative recovery of laryngeal motility[24], with Type II LOS associated with a more favourable prognosis compared to Type I LOS[25] and even signal reductions exceeding 50% are well-known to frequently correlate with the postoperative detection of VCP[16], comprehensive data regarding ISR following LOS are currently lacking, predominantly consisting of isolated case reports or small case series[15, 26–28]. Among the available studies, Schneider et al.[28] addressed traction as the most frequent cause of intraoperative stress for the ILN and identified ISR rates, whether specific precautions are taken during the surgical procedure performed by means of C-IONM. Notably, traction injuries are reported to occur more frequently in the area of Berry’s ligament: this phenomenon is likely attributed to the variability in the anatomical relationships between the ILN, Berry’s ligament, and the adjacent vascular structures[29–31].

Given these considerations, a tension-free thyroidectomy has been proposed as a strategy for minimizing traction-related nerve stress, thereby reducing the risk of intraoperative ILN functional complications[32]. Indeed, even signal reductions exceeding 50% during intraoperative monitoring are known to be associated with a high probability of postoperative VCP[16]. Conversely, the likelihood of VCP is considerably lower for signal reductions below the 50% threshold. Additionally, even minor increases in latency (greater than 10%) may potentially be associated with VCP in the postoperative setting. This latency parameter is particularly significant when C-IONM is used during surgery[16].

Clearly, in contrast to I-IONM, continuous stimulation techniques may facilitate the early detection of signal reductions, thus enabling the adoption of timely precautions to avoid complete LOS[19], although the superiority in terms of cost-effectiveness of C-IONM over I-IONM has not been exactly assessed yet[33].

Among the signal reductions exceeding 50% compared to predissection values, various risk classes have been identified basing on the magnitude of the observed signal reduction[16]. Delving deeper, electromyographic variations detected through IONM are categorized into non-threatening changes, combined events, and LOS, basing on the associated risk for injury to the ILN[16].

Although only I-IONM has been employed in our clinical practice, we meticulously considered all the events characterized by a greater than 50% reduction in vagus nerve signal compared to predissection values, particularly when a partial ISR was documented but the amplitude remained below the 50% threshold.

With regard to the outcomes of our study, we sought to identify a cut-off value for ISR following LOS, that could serve as a predictor of postoperative recovery of VCM. In detail, we found ISR ≤ 31%, between the first and the last values of signal recovery after LOS, as the minimal cut-off with a 100% sensitivity, thus serving as a predictive factor for VCP (specificity 20.8%). Youden index identified 0.837, corresponding to an ISR ≤ 13%, as the best cut-off in terms of sensitivity (94.1%) and specificity (89.6%) for VCP.

In this context, it is to underline that literature data are only partially in line with our results. Indeed, if ISR below the range of 50% to 70% after LOS is acknowledged to represent a risk factor for postoperative paralysis[9, 16], a specific intraoperative recovery threshold that may reliably predict the absence of postoperative VCP, when ISR remains below 50%, has not been defined yet. Schneider et al.[9, 16] made the effort to identify a correlation between intraoperative evidence of signal recovery and postoperative laryngeal outcomes, by detecting < 50%ISR, after LOS, as a value associated with postoperative VCP in 70%−100% of patients.

This study is not without limitations. Firstly, our final analysis is confined to patients with documented partial ISR following LOS, which inherently resulted in a relatively small sample size. Furthermore, the monocentric and retrospective design of our study introduces additional constraints. Nevertheless, these limitations are counterbalanced by our rigorous patients' selection criteria. Another potential drawback is related to the use of I-IONM rather than C-IONM, which may offer more comprehensive insights into intraoperative nerve function. Moreover, I-IONM enabled an assessment of the "amplitude" parameter but precluded the evaluation of the "latency" parameter of the vagus nerve signal.

On the other hand, the strength of our findings relies in the extensive experience of our referral center, with a caseload of more than 2000 neck procedures annually, and in the multidisciplinary management of the endocrine disease. To our knowledge, this study represents the first attempt to define a cut-off for ISR for predicting VCM status in the postoperative setting. More specifically, we aimed to provide interval values between the first and last post-dissection vagus nerve signals when LOS followed by less than 50% ISR, facilitating the estimation of postoperative laryngeal motility.

Our findings suggest that ISR may represent a reliable predictor of the full recovery of VCP. A ≥ 31% ISR is associated with normal postoperative VCM, indicating that ST could potentially be avoided in this subgroup of patients. Conversely, a ≤ 13% ISR may correlate with postoperative hypomotility. For ISR values ranging between 13 and 31%, hypomotility may or may not be present.

When an adverse event occurs on the first side during a planned TT, the decision to proceed with the surgical dissection ultimately rests with the operating surgeon, who must accurately consider the specifics of the clinical case alongside its own technical expertise.

These findings may enhance our ability to predict early postoperative cord mobility, thereby allowing for potentially avoiding further surgical trauma whether a satisfactory ISR percentage suggests normal cord function in the postoperative setting. However, larger prospective studies employing similar protocols and extended follow-up durations are still necessary to definitively validate our results.

## Data Availability

The data presented in this study are available on request from the corresponding author. The data is not publicly available due to privacy and ethical restrictions.
